# FGFR/Heartless and Smog interact synergistically to negatively regulate Fog mediated G-protein coupled receptor signaling in the *Drosophila* nervous system

**DOI:** 10.1093/g3journal/jkaa029

**Published:** 2021-03-17

**Authors:** Kumari Shweta, Anagha Basargekar, Anuradha Ratnaparkhi

**Affiliations:** MACS-Agharkar Research Institute (affiliated to SPPU, Pune), Developmental Biology Group, G.G. Agarkar Road, Pune 411 004, India

**Keywords:** heartless, Fog, *Drosophila*, Smog, Glia

## Abstract

Folded gastrulation (Fog) is a secreted ligand that signals through the G-protein-coupled receptors Mist and Smog and the G-protein Concertina to activate downstream effectors to elicit cell-shape change during gastrulation. In the embryonic central nervous system (CNS), Fog has roles in axon guidance and glial morphogenesis. However, the elements of the pathway as well as mechanisms required for transducing the signal in this context have not been determined. We find that while Concertina is essential for Fog signaling, Mist is dispensable and Smog, surprisingly, functions as a negative regulator of the pathway in the CNS. Interestingly Heartless, a fibroblast growth factor receptor, also functions as a negative regulator. Furthermore, both Heartless and Smog interact in a synergistic manner to regulate Fog signaling. Our results thus identify Heartless and Smog as part of a common regulatory pathway that functions to restrict Fog signaling in the embryonic CNS and highlights the context-specific role for Fog receptors during development.

## Introduction

Cells undergo change in shape during development to facilitate processes like cell migration, tissue extension, and tube formation, which are essential for organ formation. In *Drosophila*, G-coupled protein receptor (GPCR) signaling triggered by the ligand Folded gastrulation (Fog) brings about co-ordinated apical constriction essential for cell invagination during gastrulation (Costa *et al.* 1994). The signaling pathway consists of the Gα_12/13_ protein, Concertina (Cta; Parks and Wieschaus 1991), which in turn triggers activation of the RhoGEF2-Rho-RhoKinase (Rok) cascade leading to cell-shape change (Dawes-Hoang *et al.* 2005). GPCRs, Mist and Smog, have been identified as receptors of Fog. The gene *mist*, identified through a cell-culture based screen, is zygotically expressed in early blastoderm embryos in a pattern similar to Fog (Manning *et al.* 2013). Smog is maternally expressed and mediates part of the Fog signal during gastrulation (Kerridge *et al.* 2016). Signaling mediated by these receptors leads to apical constriction which is lost once invagination is complete. At this point, cells turn mesenchymal through activation of FGF signaling mediated by the receptor Heartless (Htl; Leptin 1999). Details of the mechanisms that lead to the downregulation of Fog signaling, a step that must precede activation of Heartless signaling are still unclear.

We are interested in understanding the mechanism of Fog signaling and its regulation in the embryonic central nervous system (CNS). Here, the expression of *fog* is detected in subset of longitudinal or interface glia (LG); in the periphery, expression is detected in scolopale and cap cells associated with the chordotonal organ (CHO, Ratnaparkhi and Zinn 2007). Knock-down of *fog* alters glial morphology leading to defects in ensheathment of the neuropil, while overexpression of *fog* leads to disorganization of the glial lattice (Ratnaparkhi and Zinn 2007). Interestingly, Htl/FGFR is also expressed in LG (Shishido *et al.* 1997) and is known to regulate the extension of glial processes into the neuropil during the embryonic and larval stages (Stork *et al.* 2014). Htl signaling is also required for glial ensheathment of photoreceptor axons (Franzdóttir *et al.* 2009); in the adult olfactory lobe, Htl is required in glia for proper compartmentalization of olfactory glomeruli (Wu *et al.* 2017).

The extent to which Fog signaling is conserved in the CNS, and its regulation in this context, is still poorly understood. In an earlier study, the orphan receptor tyrosine phosphatase PTP52F (Schindelholz *et al.* 2001), was shown to function as a positive regulator of Fog signaling. Consistent with this role, *ptp52F* mutant embryos showed presence of an irregular ventral furrow similar to *fog* mutants (Ratnaparkhi and Zinn 2007). More recently, through a wing based genetic screen, regulators of mitochondrial fusion and fission were identified as downstream modulators of Fog signaling (Ratnaparkhi 2013). The genetic interaction between *ptp52F* and *fog* suggests that pathways involving tyrosine phosphorylation, are likely to regulate Fog signaling. Supporting this, elements of the Htl signaling pathway were identified as being epistatic to *fog* in the wing screen (data not shown) carried out in our laboratory (Ratnaparkhi 2013). The overlap in expression and function of Fog and Htl signaling in LG and their common role in regulating morphogenesis, prompted us to test whether Htl might regulate Fog signaling in the CNS.

Overexpression of Fog in neurons or glia leads to ectopic axonal and glial midline crossing respectively (hereafter referred to as AMC and GMC respectively). In the current study, we have used “midline crossing” as an assay to identify elements of the Fog pathway that are essential for signaling in the CNS, and to test whether Htl regulates Fog signaling.

Our results show that Concertina is essential for Fog signaling in the CNS. In contrast, Mist does not appear to play a role in transducing the Fog signal. Interestingly, both, Htl and Smog function as negative regulators and interact in a synergistic manner to restrict the pathway. Our results thus highlight context-specific roles for Fog receptors and implicate *htl* and *smog* as part of a common genetic network involved in regulating Fog signaling.

## Materials and methods

### *Drosophila* stocks and fly husbandry

All fly stocks were raised on standard cornmeal medium. *htl^AB42^/TM3[ftz::lacZ]* (#5370), *UAS-λhtl* (#5367), *UAS*-*ctaRNAi* (#51848), *UAS*-*htlRNAi* (#35024), *C155*-GAL4 (#458), *UAS-CD4-tdGFP* (#35839), *UAS-mistRNAi* (#41930), and *UAS-htl* (#5419) are from BDSC (USA). *UAS*-*smogRNAi* (GD7852) and *UAS-mistRNAi* (GD33135) are from the VDRC Stock Center (Vienna, Austria). *htl^YY262^/TM3[ftz::lacZ]* (Gisselbrecht *et al.* 1996); *dof^111^* (M. Leptin, University of Cologne, Germany); *UAS-cta*, *UAS-cta^Q303L^*, and *cta^RC10^* (Naoyuki Fuse, NIG, Japan); *smog^KO^* and *UAS-smogC::GFP* (S. Kerridge and T. Lecuit, AMU, France); *htl*^S1–28^/TM3 (T. Kojima, Japan); *htl*-GAL4 (Alicia Hidalgo, University of Birmingham, UK); *UAS-ths::HA* (Arno Muller, UK); *UAS-ths* (Angelike Stathopoulous, Caltech, USA); *elav*-GAL4 and *W^1118^* (K. Zinn, Caltech, USA); *C155*-GAL4; and *elav*-GAL4, *UAS-fog*, and *UAS-fogRNAi* used in this study have been described previously (Ratnaparkhi and Zinn 2007). Except where stated, all experiments were carried out at 25°C. Balancers carrying lacZ or GFP were used to identify embryos of the correct genotype. The genotypes of all the lines generated and used in this study have been included as Supplementary material (Supplementary Table S1). For the experiment involving *UAS-mistRNAi^41930^*, we generated a recombinant *UAS-fog*, *UAS-mistRNAi^41930^* line. The presence of the RNAi was confirmed by staining the larval brains with anti-Mist (Supplementary Figure S1). The presence of *UAS-fog* was independently checked by staining *elav-GAL4>UAS-fog*, *UAS-mistRNAi^41930^* embryos with anti-Fog.

### Immunohistochemistry, imaging, and image analysis

Embryo fixation and immunohistochemistry were performed using standard protocols (Patel 1994). Embryos of the correct genotype were scored using lacZ or GFP balancers. The following antibodies were used: chicken anti-GFP (1:1000; Life Technologies), rabbit anti-GFP(1:1000; Life Technologies), mouse anti-GFP (1:1000; Life Technologies), rabbit anti**-**Beta Galactosidase (1:1000; Life Technologies), mouse anti-Beta Galactosidase (1:1000; Promega), mouse anti-Beta Galactosidase (1:10; DSHB, Iowa), anti-fasciclin II or mAb1D4 (1:30 or 1:50; DSHB), anti-Repo (1:10 or 1:20; DSHB), anti-Futsch (22C10, 1:100; DSHB), anti-Fog (1:500; N. Fuse, Japan), and secondary antibodies conjugated to Alexa Fluor 488, 568, or 633 (1:1000, ThermoFisher). For all immunostainings, a common cocktail containing the antibodies was made, mixed thoroughly and divided equally between control and experimental tubes. For all experiments, the samples were processed in an identical manner, at the same time, and imaged under identical confocal settings.

Images were obtained using a Leica SP8 confocal system. All glial images were obtained using a 63× objective (NA = 1.4); 40× objective (NA = 1.4) was used for all axonal images. Abdominal segments A1–A7 and A2–A7 were used to quantify AMC and GMC, respectively. ImageJ software (NIH, Bethesda) was used for image analysis. Figures were assembled using Adobe Photoshop (Version CS5).

### Geometric measurements for glia

Aspect ratio was measured using the ImageJ software (NIH). For a given glia, individual “z” sections were scanned and the section in which the glia appeared to be of maximum size was selected. Next, the outline of the cell was drawn manually using Repo (nuclear stain) and the membrane-GFP staining as markers. The “fit ellipse” and “shape descriptor” tools in ImageJ were used to obtain values for aspect ratio. For each embryo, 20 dorso-medial LG were randomly selected for analysis. The values obtained for aspect ratio were normalized to their respective control. These indices are unitless.

### Flourescence intensity measurement

Measurement of Mist intensity in Supplementary Figure S1 was done on a single optical section showing maximum staining intensity. The selection of the section was done using the plot profile function in ImageJ software. Six larval brains were analyzed for each genotype. Intensity was measured from 8 to 14 cells per brain.

### Quantitative PCR

Stage 16 and stage late 16 embryos were used to isolate total RNA using the Trizol method according to the manufacturer’s instructions. Homozygous *htl^AB42^* mutants were sorted unequivocally based on the absence of the GFP balancer and presence of gut defects associated with these mutants. cDNA synthesis was carried out from 1 µg of total RNA using Verso cDNA synthesis kit from Thermoscientific (Cat. No. AB-1453A). Quantitative PCR was carried on a QuantStudio3 (Applied Biosystems) system using SYBR Green GoTaq master mix from Promega (Cat. No. A6001). Calculation of relative gene expression was done after normalization to control *rp49* using the ΔΔC_t_ analysis method. The graph shown is the average obtained from three biological replicates. The value for each biological sample was an average of three technical replicates. The sequence of the primers used are as follows:

*smogB* Forward: 5′-GTTTCGCCTTGGGTCTGATA-3′

*smogB* Reverse: 5′-GGTCTGTGCTTATTGGTCGT-3′

*smogD* Forward: 5′-GCAGAAAAAGGGCAGCAA-3′

*smogD* Reverse: 5′-CCTCGGTGATCTCGATGT-3′

*smogF* Forward: 5′-AACCTTGAAAGCAGCCAAGA-3′

*smogF* Reverse: 5′-ATCGAACTCAAGACTGACAG-3′

*htl* Forward: 5′-CAAGCGGATCGCTGGTAGTG-3′

*htl* Reverse: 5′-GACTCCTGGCTCCCAAATGAT-3′

### Statistics

Statistical analyses were done using the GraphPad Prism Software, Version 5. Student’s *t*-test was used for analysis between two genotypes. ANOVA (Tukey’s multiple comparison test) was used for the analysis of multiple genotypes. The scatter dot plots are represented as mean ± SD. All bar graphs are mean ± SEM.

### Data availability

Strains and plasmids are available upon request. The authors affirm that all data necessary for confirming the conclusions of the article are present within the article, figures, and tables.

Supplementary material is available at figshare DOI: https://figshare.com/articles/figure/Supplemental_Material_for_Shweta_Basargekar_and_Ratnaparkhi_2020/12813299?file=25392737.

## Results

### Concertina is essential for Fog signaling in the CNS

**Figure 1:**
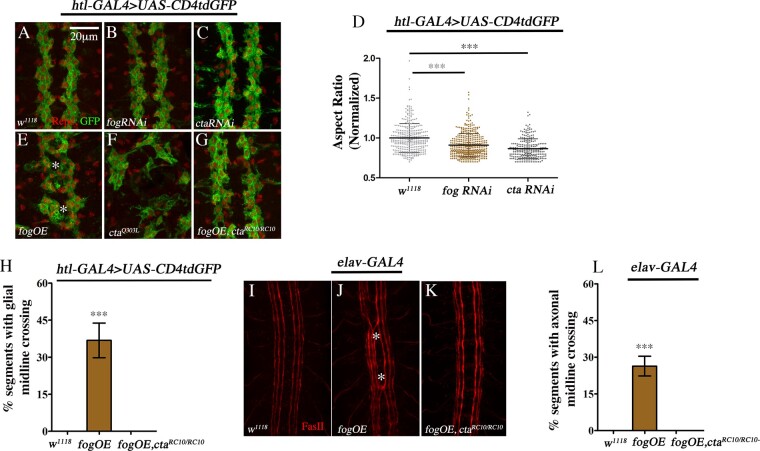
Fog regulates morphology and organization of LG. Organization and morphology of LG in the embryonic CNS of control (A), *UAS-fogRNAi* (B), and *UAS-ctaRNAi* (C). GFP (green) stains glial membrane and Repo (red) marks glial nuclei. (D) Quantification of glial aspect ratio [control: 1.0 ± 0.18 (*n* = 337) *vs UAS-fogRNAi*: 0.91 ± 0.15 (*n* = 408) *vs UAS-ctaRNAi* 0.87 ± 0.12 (*n* = 240)]. LG morphology and organization in *UAS-fog* (E), *UAS-cta^Q303L^* (F), and *UAS-fog*, *cta^RC10/RC10^* embryo (G). (H) Quantification of GMC in control (0.00, *n* = 150), *htl*-GAL4, *UAS-CD4tdGFP*>*UAS-fog* (36.81 ± 7.02%, *n* = 144), and *htl*-GAL4, *UAS-CD4tdGFP*>*UAS-fog*, *cta^RC10/RC10^* (0.00; *n* = 204). Loss of *cta* suppresses GMC completely. (I–K) CNS of late stage 16 embryo stained with anti-FasII. Control shows three distinct axon fascicles on either side of the midline (I). Ectopic axonal midline crossing (AMC, asterisk) in *elav-*GAL4*>UAS-fog* embryo (J). AMC is suppressed in a *cta* mutant (K). (L) Quantification of AMC in control (0.00, *n* = 77), *elav*-GAL4>*UAS-fog* embryos (26.37 ± 4.02%, *n* = 182), and *elav*-GAL4>*UAS-fog*, *cta^RC10/RC10^* (0.00, *n* = 252). Error bar in (D) represents SD; in (H) and (L), the error bar represents SEM. One-way ANOVA with post hoc Tukey’s test. ***P ≤ 0.001. *n* = number of segments.

In the embryonic CNS, expression of *fog* is enriched in a subset of LG and downregulation of the gene leads to LG becoming smaller and more spherical in shape (Ratnaparkhi and Zinn 2007; compare Figure 1, A and B). We knocked down *concertina* in glia using RNAi (*ctaRNAi*) and found that it exerts a similar effect: the LG were smaller in size and more spherical (Figure 1C). A measurement of the aspect ratio revealed a small but significant decrease of 13% compared to control (Figure 1D).

Overexpression of *fog* in glia leads to disorganization of the glial lattice, and embryonic lethality (Ratnaparkhi and Zinn 2007). A closer look at the disorganization revealed that in some segments, LG are present either at the midline or closer to the midline with processes extending across (Figure 1E, asterisk). We refer to this phenotype as ectopic “glial midline crossing” or GMC. In terms of morphology, the glia appeared stretched and more tightly packed. Interestingly, overexpression of *concertina* did not significantly alter glial organization; however, expression of a constitutively active Concertina (*UAS-cta^Q303L^*/*UAS-cta^CA^*) led to severe defects and embryonic lethality: LG appeared scattered and stretched. In addition, the CNS in these embryos failed to condense (Figure 1F).

We carried out genetic epistasis experiments to determine whether Concertina is essential for Fog signaling using GMC as an assay. In *htl*-GAL4>*UAS-fog* embryos, 37% of the segments exhibit GMC. This phenotype was completely suppressed in a *cta^RC10/RC10^* mutant background (Figure 1, G and H).

To corroborate the above results, we conducted the experiment by overexpressing *fog* in neurons which leads to ectopic axonal midline crossing (AMC, Ratnaparkhi and Zinn 2007). In control or wild-type embryos, anti-Fasciclin II stains 3 axon bundles on either side of the midline (Figure 1I). Pan-neuronal overexpression of *fog* leads to misrouting of these axons across the midline (Figure 1J, asterisk) in ∼26% of the segments. As with glia, AMC was completely suppressed in a homozygous *cta* mutant background (Figure 1, K and L). Together, these results indicate that Concertina is essential for Fog signaling in the embryonic CNS.

The above results also highlight the strong similarity between glia and neurons in their response to Fog overexpression, implying that results from one can be extended to the other. This, coupled with the ease with which one can score AMC, prompted us to choose this as an assay to evaluate all subsequent genetic interactions.

### Smog functions as a negative regulator of Fog signaling in the CNS

Fog binds to GPCRs Mist and Smog (Manning *et al.* 2013; Kerridge *et al.* 2016). ModENCODE data show that the latter is expressed at high levels and almost exclusively in the CNS (www.flybase.org). Based on this, we sought to first test if Smog functions as a receptor for Fog in the CNS.

**Figure 2:**
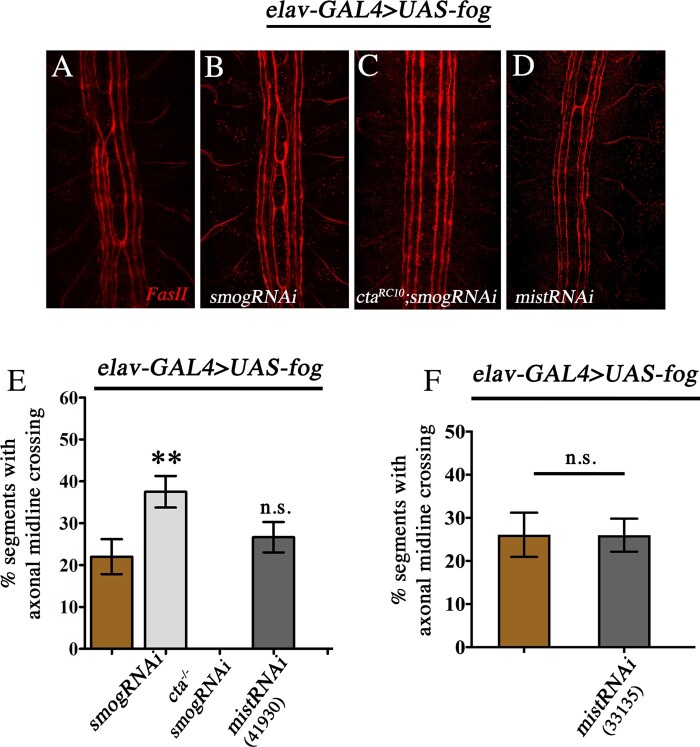
Smog is a negative regulator of Fog signaling in the CNS. (A–D) CNS stained with anti-FasII (red). AMC in *elav*-GAL4>*UAS-fog* (A); *elav*-GAL4>*UAS-fog*, *UAS*-*smogRNAi* (B); *elav*-GAL4>*UAS-fog*, *UAS-smogRNAi*, *cta^RC10/RC10^* (C), and *elav*-GAL4>*UAS-fog*, *UAS-mistRNAi* (D). (E) Percentage of AMC in *elav*-GAL4>*UAS-fog* (22.01 ± 4.16, *n* = 259); *elav*-GAL4>*UAS-fog*, *UAS-smogRNAi* (37.05 ± 3.78, *n* = 224); *elav*-GAL4>*UAS-fog*, *UAS-smogRNAi cta^RC10RC10^* (0, *n* = 259); and *elav*-GAL4>*UAS-fog*, *UAS-mistRNAi^41930^* (26.64 ± 3.64, *n* = 259). (F) Percentage of AMC in *elav*-GAL4>*UAS-fog* (26.09 ± 5.13, *n* = 161) and *elav*-GAL4>*UAS-fog*, *UASmistRNAi^33135^* (25.97 ± 3.83, *n* = 154). One-way ANOVA with post hoc Tukey’s test. *P* ≤ 0.01(**), *P* > 0.05 = not significant (n.s.). *n* = number of segments.

We used genetic epistasis to test this possibility and reasoned that if Smog functions as the Fog receptor, then AMC due Fog overexpression would be suppressed upon downregulation of *smog*. However, surprisingly, we observed a significant increase in AMC. Compared with *elav*-GAL4>*UAS-fog* (Figure 2, A and E), embryos co-expressing *UAS-smogRNAi* showed a near 70% increase in midline crossing (Figure 2, B and E) which was completely suppressed in the absence of *cta* (*elav*-GAL4>*UAS-fog*, *cta^RC10/RC10^*; *UAS-smogRNAi*; Figure 2, C and E) indicating that the increase is indeed due to upregulation of the Fog signaling. We ruled out the possibility of additive effects arising from expression of *UAS-smogRNAi*, as we do not observe any midline crossing upon expression of *UAS-smogRNAi* or in *smog^KO/KO^* embryos (data not shown).

Given the unexpected result with *smog*, we sought to test whether Mist might function as the Fog receptor in the CNS. Interestingly, knock-down of *mist* did not alter the frequency of AMC (compare Figure 2, A and D) or GMC (Supplementary Figure S2). The percentage of segments with AMC in *elav*-GAL4>*UAS-fog*, *UAS*-*mistRNAi^41930^* embryos was comparable to *elav*-GAL4>*UAS-fog* (Figure 2E). This result was further confirmed through use of a second, independent *UAS-mistRNAi* line (Figure 2F), leading us to conclude that Mist is unlikely to function as a Fog receptor in the CNS.

Together, the above findings reinforce the role of Concertina (Gα_12/13_) as an essential component of the Fog pathway and indicate that, in the context of the CNS, Smog functions as a negative regulator to restrict Fog signaling.

### *Heartless* negatively regulates Fog signaling

Htl is expressed in glia and regulates glial morphogenesis in the embryo (Shishido *et al.* 1997; Stork *et al.* 2014). To determine whether *fog* and *htl* interact, we first examined LG organization in *htl* mutants to determine the extent of similarity, if any, to *fog* loss-of-function and gain-of-function embryos. *Htl^AB42^* is a null mutant for the gene (Gisselbrecht *et al.* 1996) and in homozygous mutant embryos LG appear slightly disorganized, small and more clustered with occasional breaks in the glial lattice (compare Supplementary Figure S3, A and B). The tendency to cluster seemed more reminiscent of *fog* overexpression than *fogRNAi* or *cta* mutants (compare Supplementary Figure S3B with Figure 1E). A similar phenotype was also observed in *htl-*GAL4, *UAS-CD4tdGFP*>*UAS-htlRNAi* embryos (Supplementary Figure S3C). Interestingly, overexpression of *UAS-λhtl* (constitutively active) and *UAS-thisbe* did not alter organization, size, or aspect ratio of LG. The absence of a phenotype was not due to the lack of expression of the transgene because, as previously reported (Avet-Rochex *et al.* 2012; Avet-Rochex *et al.* 2014), overexpression of *UAS-λhtl* using *repo*-GAL4 led to a robust proliferation of glia in the 3rd instar larval brain (data not shown).

**Figure 3:**
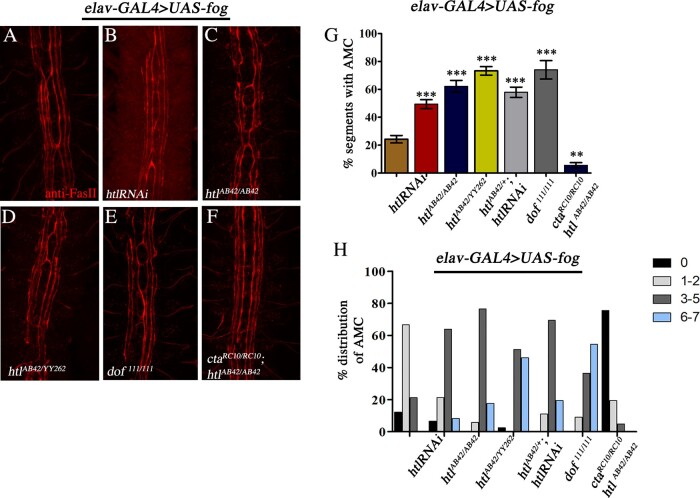
Loss of *htl* enhances AMC caused by Fog overexpression. (A–F) Embryonic CNS stained with anti-FasII (red). AMC in various mutant backgrounds is shown. (G) Quantification of AMC in *UAS-fog* (control) (24.24 ± 2.67, *n* = 231); *htlRNAi* (49.41 ± 3.16, *n* = 427); *htl^AB42/AB42^* (62.18 ± 4.23, *n* = 119); *htl^AB42/YY262^* (73.26 ± 3.07, *n* = 273); *htl^AB42/htlRNAi^* (57.93 ± 3.69, *n* = 252); *dof^111/111^* (74 ± 6.62, *n* = 77); and *cta^RC10/RC10^*; *htl^AB42AB42^* (5.58 ± 1.92, *n* = 287). (H) Percentage distribution of AMC in *UAS-fog* (control; *N* = 33; 0 : 12.12; 1–2:  66.66; 3–5: 21.21; 6–7: 0); *UAS-fog;UAS-htlRNAi* (*N* = 61; 0: 6.56; 1–2: 21.31; 3–5: 63.93; 6–7: 8.2); *UAS-fog;htl^AB42/AB42^* (*N* = 17; 0: 0; 1–2: 5.88; 3–5: 76.47; 6–7: 17.65); *UAS-fog htl^AB42/YY262^* (*N* = 39; 0: 2.56; 1–2: 0; 3–5: 51.28; 6–7: 46.15); *UAS-fog;htl^AB42/+^*, *UAS-htlRNAi* (*N* = 36; 0: 0; 1–2: 11.11; 3–5: 69.44; 6–7: 19.44); *UAS-fog;dof^111/111^* (*N* = 11; 0: 0; 1–2: 9.09; 3–5: 36.36; 6–7: 54.54); *UAS-fog*, *cta^RC10/RC10^;htl^AB42/AB42^* (*N* = 41; 0: 75.6; 1–2: 19.5; 3–5:  4.5; 6–7:  0). One-way ANOVA with post hoc Tukey’s test. *P* ≤ 0.001(***), *P* ≤ 0.01(**). *N* = number of embryos; *n* = number of segments.

Next, we overexpressed *fog* in neurons and used AMC as an assay to test for interaction between *fog* and *htl.* Interestingly, co-expression of *UAS-htlRNAi* with *UAS-fog* led to a near 2-fold increase (49%) in AMC (compare Figure 3, A, B, and G). A significant increase was also observed in the background of *htl^AB42/AB42^* and *htl^AB42/YY262^* mutants resulting in 62% and 73% midline crossing, respectively (Figure 3, C, D, and G). To rule out possible additive effects, we examined axonal organization in *htl^AB42/AB42^* and *htl^YY262/YY262^* embryos in the absence of *fog* overexpression. In both mutants, AMC was present at a very low frequency of approximately 6% and 1.2%, respectively (Supplementary Figure S4). Furthermore, in *htl^AB42/YY2262^*, the frequency of AMC was 1.6% (data not shown).

Taken together, this indicates that the effect of *htl* on AMC is indeed due to a synergistic interaction with *fog* and suggests that Htl functions as a negative regulator of Fog signaling.

*Stumps*/*downstream-of-fgf* (*dof*) is a specific effector of FGFR signaling in *Drosophila* (Vincent *et al.* 1998; Michelson *et al.* 1998; Imam *et al.* 1999). Consistent with the above results, loss of *dof^111/111^* also led to an increase in AMC in a manner comparable to *htl* mutants: 74% of the segments showed midline crossing (Figure 3, E and G) indicating that the pathway mediated by Htl, and not just the receptor, regulates Fog signaling.

We confirmed that the increase in AMC observed in *htl* mutants is due to the upregulation of Fog signaling, by expressing *UAS*-*fog* in a *cta* and *htl* double mutant (*cta^RC10/RC10^;htl^AB42/AB42^*). Midline crossing was strongly suppressed in these embryos (Figure 3, F and G): AMC was observed in only 5.6% of the segments, a frequency that is observed in homozygous *htl^AB42^* mutants in the absence of *fog* overexpression.

We further analyzed the nature of the enhancement by measuring the distribution of embryos with 0, 1–2, 3–5 and 6–7 cross-overs in the different genotypes. As shown in Figure 3H, approximately 60% and 20% of *elav*-GAL4>*UAS-fog* embryos belonged to the category of 1–2 and 3–5 AMC, respectively, with none in the 6–7 category. In contrast, loss of *htl* was seen to increase the percentage of embryos with 3–5 AMC with some in the 6–7 category as well. This was particularly striking in the background *htl^AB42/YY262^* and *dof^111^* mutants where nearly 50% of the embryos are seen to fall into the category of 6–7 AMC (Figure 3H). AMC was significantly suppressed in *cta^RC10/RC10^;htl^AB42/AB42^* double mutant background with the majority of the embryos displaying complete absence of midline crossing.

Based on the above results, we wondered whether expression of constitutively active *htl* (*UAS-λhtl*) might suppress the AMC phenotype. Interestingly, co-expression of *UAS-fog* with *UAS-λhtl* led to a significant increase in AMC (Supplementary Figure S5, B and E). A similar enhancement was observed upon co-expression with *UAS-thisbe::HA* (*ths::HA*) and *UAS-htl*, although in case of the latter, the extent of increase seemed less compared with the others (Supplementary Figure S5, C and E). Notably, individual expression of neither *UAS-λhtl*, *UAS-htl*, nor *UAS-ths::HA* resulted in AMC (Supplementary Figure S5E). In case of *ths*, we examined both sibling embryos lacking *UAS-fog* (*elav*-GAL4/CyolacZ; *UAS*-*ths*::HA) and *C155-*GAL4*;elav-*GAL4*>UAS-ths* embryos. In both cases, we failed to detect midline crossing (Supplementary Figure S4E), ruling out additive effects. Thus, both *htl* loss of function and gain of function enhance Fog signaling indicating the presence of a signaling threshold for modulation of the pathway.

### The interaction between Htl and Fog is dependent on coincident signaling

**Figure 4:**
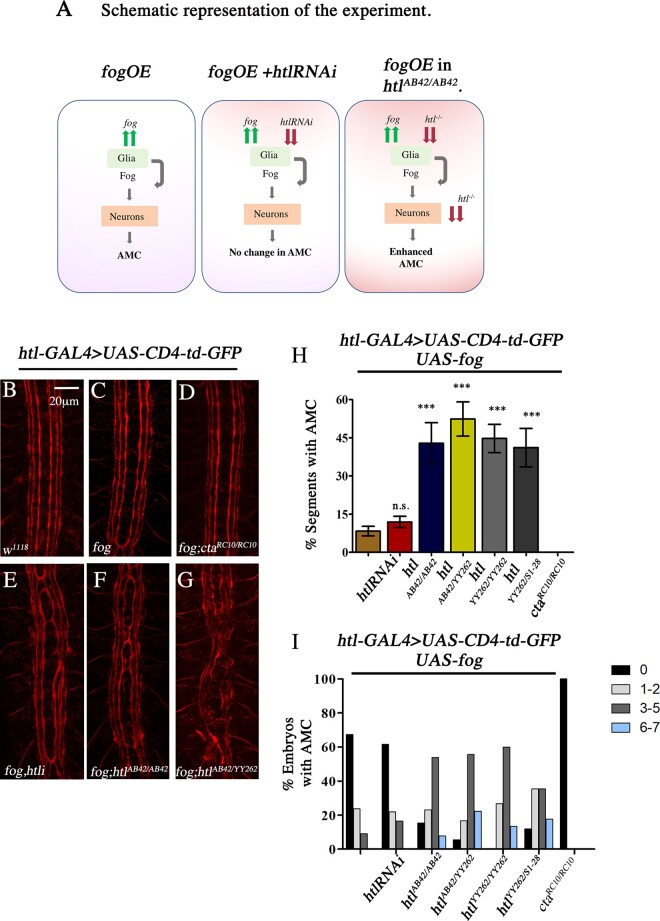
Coincident Htl activity is essential for regulation of Fog signaling. (A) Schematic representation of the experiment. (B–G) Embryonic CNS stained with anti-FasII (1D4) showing AMC in response to glial overexpression of *UAS-fog* in different mutant backgrounds. (H) Quantification of AMC: *UAS-fog* (control, 8.31 ± 1.88, *n* = 385); *UAS-fog;UAS-htlRNAi* (11.94 ± 2.23, *n* = 511); *UAS-fog;htl^AB42/AB42^* (42.86 ± 8.09, *n* = 91); *UAS-fog;htl^AB42/YY262^* (52.38 ± 6.73, *n* = 126); *UAS-fog;htl^YY262/YY262^* (44.76 ± 5.53, *n* = 105); *UAS-fog;htl^YY262/S1–28^* (41.18 ± 7.54, *n* = 119); and *UAS-fog*, *cta^RC10/RC10^* (0.00, *n* = 112). (I) Percentage distribution of AMC: *UAS-fog* (*N* = 55; 0: 67.27; 1–2: 23.64; 3–5:  9.09; 6–7: 0); *UAS-fog;UAS-htlRNAi* (*N* = 73; 0: 61.64; 1–2: 21.92; 3–5: 16.44; 6–7: 0); *UAS-fog;htl^AB42/AB42^* (*N* = 13; 0: 15.38; 1–2: 23.08; 3–5: 53.85; 6–7: 7.69); *UAS-fog;htl^AB42/YY262^* (*N* = 18; 0: 5.56; 1–2: 16.67; 3–5: 55.56; 6–7: 22.22); *UAS-fog;htl^YY262/YY262^* (*N* = 15; 0: 0; 1–2:26.61; 3–5: 60; 6–7: 13.3); *UAS-fog;htl^S1–28/YY262^* (*N* = 17; 0: 11.76; 1–2: 35.29; 3–5: 35.29; 6–7:  17.65); and *UAS-fog*, *cta^RC10/RC10^* (*N* = 16; 0:  100). *N* = number of embryos; *n* = number of segments. One-way ANOVA with post hoc Tukey’s test. *P* ≤ 0.001(***), *P* > 0.05 not significant (n.s.).

Next, to determine the manner in which the two pathways interact, we took advantage of the fact that Fog is extracellular and that its overexpression in glia can activate signaling in neurons leading to AMC (Ratnaparkhi and Zinn 2007). We reasoned that if Htl influences Fog signaling in a “cell autonomous” manner, then co-expression of *UAS-fog* with *UAS*-*htlRNAi* in glia alone should not alter the frequency of AMC, as loss of *htl* in glia would be unable to influence Fog signaling activated in neurons. However, the same experiment when conducted in an *htl* mutant would enhance midline crossing (Figure 4A).

In *htl*-GAL4, *UAS*-*CD4-tdGFP*>*UAS*-*fog* embryos, AMC is observed at a rather low frequency (compare Figure 4B with Figure 4C) of approximately 8% (Figure 4H). This phenotype is completely suppressed in *concertina* mutant background (*htl*-GAL4, *UAS-CD4-tdGFP*>*UAS*-*fog*, *cta^RC10/RC10^*; Figure 4D). Consistent with our hypothesis, embryos expressing both *UAS-fog* and *UAS-htlRNAi* in glia (*htl*-GAL4, *UAS-CD4-tdGFP*>*UAS*-*fog;UAS-htlRNAi*) did not exhibit any increase in AMC. The frequency was approximately 12% (Figure 4, E and H) and the distribution of midline crossing in both genotypes was similar (Figure 4I). In contrast, expression of *UAS-fog* in *htl^AB42/AB42^* (Figure 4F) and *htl^AB42/YY262^* mutants (Figure 4G) led to a sharp increase in AMC (42% and 52%, respectively; Figure 4H) with a dramatic increase in the number of embryos with three or more AMCs (Figure 4I). This indicates that Htl can influence Fog signaling only if the two pathways function in the same cell, thus ruling out non-cell autonomous effects.

### *Htl* and *smog* interact in a synergistic manner to regulate Fog signaling

**Figure 5:**
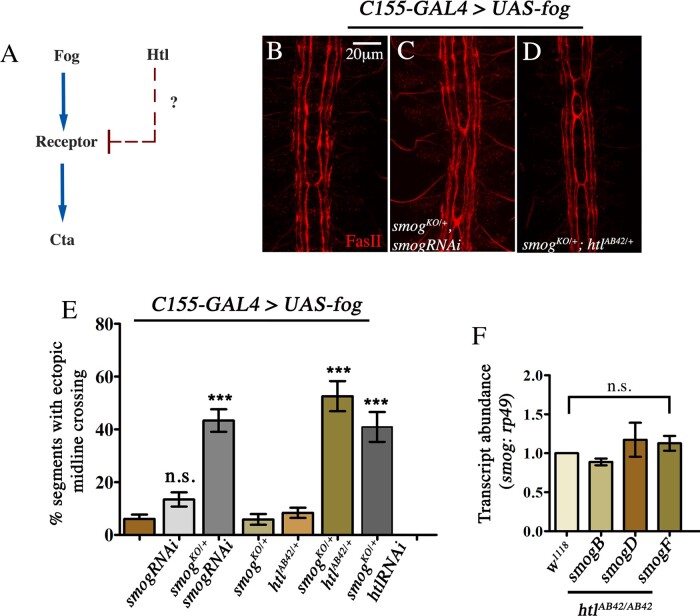
*Smog* and *htl* interact in a synergistic manner to regulate Fog signaling. (A) The interaction between Fog and Htl signaling pathways predicts that the regulation is likely to be at the level of the receptor. Embryonic CNS stained with anti-FasII (red) showing AMC in *UAS-fog* (B), *UAS-fog*, *smog^KO/+^*, *UAS-smogRNAi* (C) and *UAS-fog*, *smog^KO/+^*, *htl^AB42/+^* (D) mutants. (E) Quantification of AMC: *UAS-fog* (control, 6.06 ± 1.65%, *n* = 231)*; UAS-smogRNAi* (13.45 ± 2.69%, *n* = 238); *smog^KO/+^;UAS-smogRNAi* (43.35 ± 4.28%, *n* = 203); *smog^KO/+^* (5.8 ± 2.03%, *n* = 154); *htl^AB42^*^/+^ (8.36 ± 1.93%, *n* = 287); *smog^KO^*^/+^*;htl^AB42^*^/+^ (52.57 ± 5.7%, *n* = 175); and *UAS-fog*, *smog^KO^*^/+^*;UAS-htlRNAi* (40.91 ± 5.66%, *n* = 154). (F) Normalized transcript levels of *smogB*, *smogD*, and *smogF* in *htl^AB42/AB42^* mutants (*w^1118^*: 1.0; *smogB*: 0.89 ± 0.04; *smogD*: 1.17 ± 0.22; *smogF*: 1.13 ± 0.10). The values shown in (E) and (F) are mean ± SEM. One-way ANOVA with post hoc Tukey’s test. *P* < 0.05. *n* = number of segments. ***P ≤ 0.001, *P* ≥ 0.05 = not significant (n.s.).

How does Htl regulate Fog signaling? The observation that downregulation of *htl* enhances AMC due to Fog overexpression, and that this can be completely suppressed by loss of Cta (Figure 3G), suggests that the site of regulation is likely to be the receptor (Figure 5A). Since both Htl and Smog function as negative regulators of the pathway, we wondered whether the two genes might interact to regulate signaling. To test this, we used *C155*-GAL4 to drive expression *UAS*-*fog*. This gives rise to a weak AMC phenotype with a frequency of approximately 6% (Figure 5, B and E). Consistent with the earlier result, knock-down of *smog* using RNAi led to a small increase in AMC (13%; Figure 5E); a stronger enhancement of the phenotype was observed upon expression of *UAS*-*smogRNAi* in a *smog^KO/+^* mutant (43%; Figure 5, C and E).

**Figure 6:**
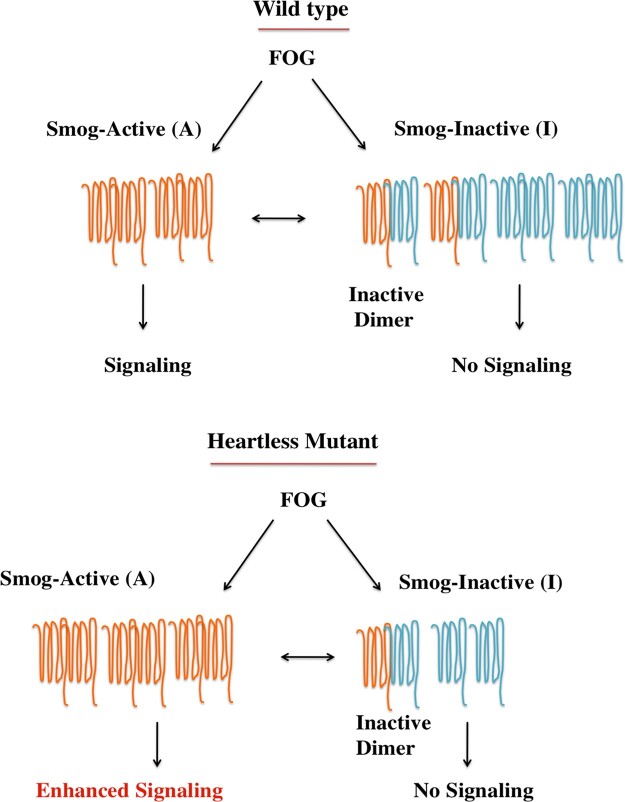
Proposed model to explain regulation of Fog signaling by Htl. We hypothesize that an inactive variant of Smog (Smog-I) is expressed in the CNS. Smog-I binds Fog but does not transduce the signal. Dimers with Smog-I are thus inactive. Htl signaling helps maintain a dynamic equilibrium between Smog-I and “active” Smog (Smog-A). Absence or downregulation of Htl signaling destabilizes Smog-I, thus making more ligand available for signaling and shifting the equilibrium toward Smog-A, leading to enhanced signaling.

To determine if *htl* and *smog* interact, we expressed *UAS-fog* in embryos that were heterozygous for both genes (*smog^KO^/+;htl^AB42^/+*) and scored for AMC in these animals. Interestingly, while neither *smog^KO/+^* nor *htl^AB42/+^* had any significant effect on midline crossing (6% and 8%, respectively; Figure 5E), a dramatic increase was observed in double heterozygous (*smog^KO/+^;htl^AB42/+^*) mutant embryos with 53% of the segments exhibiting AMC, a frequency comparable to *elav*-GAL4>*UAS-fog;htl^AB42/AB42^* embryos (Figure 5, D and E; compare Figures 3G and 5E). An increase in AMC was also observed in *C155*-GAL4>*UAS-fog*, *Smog^KO/+^;UAS-htlRNAi* embryos (Figure 5E). Furthermore, in the absence of *fog* overexpression, no AMC was observed in *smog^KO/+^;htl^AB42/+^* embryos (data not shown). Together, these results indicate that *htl* and *smog* interact synergistically to regulate Fog signaling.

Based on the above results we checked if Htl regulates *smog* expression by measuring *smog* mRNA levels in wild-type and *htl^AB42^* mutant embryos using quantitative RT-PCR (qPCR).

*Smog* encodes multiple splice variants that differ from each other at the C-terminus. Of these, *smogF* and *smogB* represent the longest and shortest variant respectively, with the latter having a short and unique C-terminal domain. Using transcript-specific primers we measured the relative abundance of *smog B*, *D*, and *F* in wild-type embryos and checked if their levels were altered in *htl* mutants. Of the three splice variants, *smogF* was found to be the most abundant (Supplementary Figure S6C). Furthermore, expression of these variants was not significantly altered in *htl^AB42/AB42^* mutants (Figure 5F). As a control, we measured and compared the decrease in *htl* expression between wild-type and *htl^AB42/AB42^* mutants and found a 70% reduction in transcript level (Supplementary Figure S6D). This indicates that *htl* does not regulate *smog* transcription and suggests that the interaction between the two genes is likely to be indirect, possibly via a posttranscriptional mechanism. Together, these results show that, in the embryonic CNS, Htl and Smog function as part of regulatory network to restrict Fog signaling.

## Discussion

Fog activates one the earliest zygotic signaling pathways during development to co-ordinate cell shape for invagination of the presumptive mesoderm (Costa *et al.* 1994). It is also known to play a role in the development of the salivary glands, the adult wing, axon guidance and glial morphogenesis in the embryonic CNS (Lammel and Saumweber 2000; Nikolaidou and Barrett 2004; Ratnaparkhi and Zinn 2007). The extent to which this signaling pathway is conserved in these different developmental contexts and its mode of regulation needs to be understood. We have addressed some of these questions in the context of the embryonic CNS.

It is established that Concertina is essential for Fog signaling during gastrulation (Morize *et al.* 1998). Our study shows that this holds good in the context of the CNS as well, given that both AMC and GMC due to Fog overexpression are completely suppressed in a *concertina* mutant (Figure 1). In addition, the lethality caused by Fog overexpression in glia is also suppressed.

A key finding here is the interaction between *fog* and *htl* which signal to regulate morphogenesis, first during gastrulation (Leptin 1999) and later, in the LG (Shishido *et al.* 1997; Stork *et al.* 2014). The role of Htl signaling appears to be context dependent. In the embryo, consistent with the findings of Stork and colleagues (Stork *et al.* 2014), we do not find any change in the number of Repo and Prospero positive LG in *htl* mutants. Further, expression of *UAS-λhtl* in glia does not affect glial number either. This suggests that in the embryo, the role of Htl in the glia is primarily associated with morphogenesis and not proliferation or differentiation. However, in the larva, expression of *UAS-λhtl* leads to glial proliferation (Avet-Rochex *et al.* 2012) and mRNA levels of *fog* and *smog* appear to be altered as well (Avet-Rochex *et al.* 2014), not only supporting our findings regarding an interaction between the Fog and Htl signaling but also suggesting a context dependent mode of regulation.

It is curious that even though Htl functions as a negative regulator of Fog signaling, the mutants do not exhibit any of the strong CNS phenotypes associated with Fog overexpression. The only resemblance to it is seen in the clustering of glia—a phenotype also seen in embryos expressing *UAS-fog*. This suggests that Fog signaling is likely to be tightly regulated and Htl is probably one of the many regulators of this pathway.

The interaction between Fog and Htl signaling pathways appears to be through Smog, a GPCR known to bind Fog (Kerridge *et al.* 2016). Our results show that *smog* functions as a negative regulator of Fog signaling in the CNS. In addition, loss of one copy each of *htl* and *smog* leads to a synergistic enhancement of Fog signaling clearly indicating that they are part of the same regulatory pathway (Figure 5).

How does one reconcile the interaction between Htl and Smog with the role of Smog as a negative regulator of Fog signaling in the CNS, and its function as a Fog receptor during gastrulation? The answer to this conundrum may lie in the fact that *smog* encodes multiple splice variants which, except for differences in the C-terminal region, are otherwise identical (Supplementary Figure S6). It is possible that the CNS expresses at least two isoforms of Smog which form homo and heterodimers wherein the active dimer binds and activates Fog signaling while the inactive dimer binds and sequesters Fog thus blocking signaling.

We hypothesize that Htl regulates the dynamic equilibrium between these two dimeric states by regulating the levels of one of the isoforms (Smog-Inhibitory/Smog-I) that gives rise to the inactive state. That GPCRs function as dimers is established (Bai 2004; Milligan 2004) and a recent study by Jha and colleagues has shown that Fog triggers oligomerization of Smog (Jha *et al.* 2018). It is possible that loss of Htl affects the homeostasis of Smog-I leading to downregulation of this isoform, which effectively increases not only the number of “active receptors” but also the amount of available ligand, resulting in enhanced signaling (Figure 6). In contrast, constitutive activation of Htl, might function by stabilizing the transducing receptor complex that enables prolonged signaling. The mode for such a regulation in both cases could be phosphorylation.

Phosphorylation by GPCR receptor kinases (Gprks) and other kinases followed by recruitment of beta-arrestins is one of the keys steps in GPCR inactivation and endocytosis (DeWire *et al.* 2007; Gurevich and Gurevich 2019). Recent studies on rhodopsin show that phosphorylation patterns on GPCRs can be grouped according to their ability to bind and stabilize, activate, modulate and even inhibit beta-arrestin binding (Mayer *et al.* 2019). It is therefore conceivable that Htl regulates Smog stability through such a mechanism. Consistent with this, an analysis of the predicted phosphorylation sites in the different Smog isoforms shows presence of a unique pattern in each variant (Supplementary Figure S7).

In the model described above, the regulation by Htl would necessarily be context dependent, based on the identities and expression level of the *smog* isoforms. The fact that variants Smog B, D, and F are differentially expressed in the embryo (Supplementary Figure S6) lends support to this possibility.

It has been difficult to dissect the expression pattern of individual isoforms in the embryo using in situ given the strong nucleotide sequence identity amongst the splice variants. *SmogC*, which encodes one of the longer isoforms of *smog*, has been shown to bind and mediate the Fog signal during gastrulation (Kerridge *et al.* 2016). Supporting this, expression of *UAS*-*smogC::GFP* in the CNS enhances the AMC due to Fog overexpression (Supplementary Figure S5). Based on sequence comparison of all the isoforms, we predict that SmogB could function as a potential negative regulator given its very short C-terminal domain. However, this will need to be tested.

Our results thus suggest a role for FGFR/Htl in modulating and fine-tuning Fog signaling in a threshold dependent manner. At a low signaling threshold, Htl could potentiate Fog signaling by affecting the stability of the negative regulator whereas, at a high threshold, it could enhance signaling by stabilizing the signaling complex. Whether this is indeed so, will need to be tested through future studies.
