# Corrigendum to: FGFR/Heartless and Smog interact synergistically to negatively regulate Fog mediated G-protein coupled receptor signaling in the *Drosophila* nervous system

**DOI:** 10.1093/g3journal/jkab292

**Published:** 2021-09-18

**Authors:** Kumari Shweta, Anagha Basargekar, Anuradha Ratnaparkhi

*G3 Genes|Genomes|Genetics*, 2021, 11(3)., DOI: https://doi.org/10.1093/g3journal/jkaa029

When this paper first published, in the data availability section, the location of supplementary data was erroneously given as “figshare DOI: https://doi.org/10.25387/g3.12813299”. This should have been “https://figshare.com/articles/figure/Supplemental_Material_for_Shweta_Basargekar_and_Ratnaparkhi_2020/12813299?file=25392737”. This has now been corrected online.

